# ALKBH5 inhibited autophagy of epithelial ovarian cancer through miR-7 and BCL-2

**DOI:** 10.1186/s13046-019-1159-2

**Published:** 2019-04-15

**Authors:** Hongtao Zhu, Xiaoling Gan, Xingwei Jiang, Shuai Diao, Huan Wu, Jianguo Hu

**Affiliations:** Department of Obstetrics and Gynecology, Second Affiliated Hospital, Chongqing Medical University, Linjiang Road, No. 76, Chongqing, 400010 China

**Keywords:** ALKBH5, BCL-2, Beclin1, Autophagy, miR-7, EGFR

## Abstract

**Background:**

ALKBH5 regulated the malignant behavior of breast cancer and glioblastoma. However, the expression and function of ALKBH5 in epithelial ovarian cancer have not yet been determined. In the present study, we investigated the expression and function of ALKBH5 in epithelial ovarian cancer with respect to its potential role in the tumorigenesis of the disease as well as an early diagnostic marker.

**Methods:**

Immunohistochemistry and western blot were used to detect protein expression. Gene silencing and over-expression experiment were used to study gene function. Cell proliferation assay and Matrigel invasion assays were used to detect cell proliferation and invasion, respectively. The nude mouse tumor formation experiment was used to evaluate the growth of cells in vivo.

**Results:**

The expression of ALKBH5 was found to be increased in epithelial ovarian cancer tissue as compared to the normal ovarian tissues. The silencing of ALKBH5 in SKOV3 cells enhanced the autophagy and inhibited the proliferation and invasion in vitro and in vivo, whereas the ectopic expression of ALKBH5 in A2780 cells exerted an opposite effect. Mechanical study revealed that ALKBH5 physically interacted with HuR. ALKBH5 activated EGFR-PIK3CA-AKT-mTOR signaling pathway. Also, ALKBH5 enhanced the stability of BCL-2 mRNA and promoted the interaction between Bcl-2 and Beclin1.

**Conclusion:**

Overall, the present study identified ALKBH5 as a candidate oncogene in epithelial ovarian cancer and a potential target for ovarian cancer therapy.

**Electronic supplementary material:**

The online version of this article (10.1186/s13046-019-1159-2) contains supplementary material, which is available to authorized users.

## Background

ALKBH5 is an N6-methyladenosine (m6A) eraser protein. It is expressed high in the testes but less in the heart and brain. ALKBH5 effectuates nuclear RNA export and metabolism, gene expression. ALKBH5 was also involved in mouse and human fertility [1–3]. In the hypoxia condition, the expression of ALKBH5 was increased. Also, HIF-1α elevated the expression of ALKBH5 via interaction with the promoter, in turn, promoting the transcription of ALKBH5. In breast cancer, ALKBH5 mediated the m6A-demethylation of *NANOG* mRNA, thereby inducing the breast cancer stem cell phenotype [4, 5]. ALKBH5 is highly expressed in glioblastoma stem-like cells (GSCs), and the downregulation of expression inhibited the proliferation of patient-derived GSCs. ALKBH5 mediated the m6A-demethylation of *FOXM1* mRNA, leading to enhanced FOXM1 expression [6]. However, the expression and function of ALKBH5 were not elucidated in epithelial ovarian cancer.

In the present study, we elucidated the expression of ALKBH5 and its potential clinical significance in epithelial ovarian cancer, in order to clarify the putative function of ALKBH5 in malignancy, progression, and prognosis of cancer.

## Materials and methods

### Tissue specimens

The tissue microarray slides containing malignant and normal ovarian tissues (*n* = 158) were purchased from US Biomax. The Ethics Committee of the Chongqing Medical University approved the study protocols and the use of archived cancer tissues.

### Immunohistochemistry (IHC)

IHC was performed as described previously. The antigen of the tissues was retrieved after dewaxing and hydration, followed by heating in citrate buffer (Sigma-Aldrich, PBS1). The slides were treated with 3% hydrogen peroxide (ZSGB-BIO ORIGENE, SP-9000) for 15 min to block the endogenous peroxidase activity, and then, incubated with 5% goat serum at room temperature for 30 min (Bioss Biotechnology Company, C-0005). Next, the tissues were probed with primary antibody against ALKBH5 (ab234528, Abcam, 1:100) overnight at 4 °C. For negative controls, irrelevant primary antibodies were applied. The corresponding secondary antibodies, conjugated to horseradish peroxidase (ZSGB-BIO ORIGENE, SP-9000), were incubated with the sections for 1 h at room temperature. After washing with PBS, the sections were incubated in horseradish enzyme-labeled chain avidin solution for 30 min at 37 °C and washed again. Finally, the proteins were visualized by diaminobenzidine (ZSGB-BIO ORIGENE, ZLI-9017). The staining intensity was graded on a 0–3 scale as follows: 0, absence of staining; 1, weakly stained; 2, moderately stained; 3, strongly stained. The percentage of positive tumor cells was scored as follows: 0, absence of tumor cells; 1, < 33% of tumor cells; 2, 33–66% of tumor cells; 3, > 66% of tumor cells. The IHC score (0–9) was calculated by multiplying the intensity and the percentage scores [7].

### Cell culture and transfection

Human ovarian cancer cells (A2780, SKOV3, OVCAR-3 and COC1) were cultured in RPMI 1640 medium (Sigma-Aldrich, R8758) containing 10% fetal bovine serum (FBS) and antibiotics. The cells were incubated under 5% CO_2_ at 37 °C. The third generation of cells was used to perform experiment. The double-strand oligonucleotides corresponding to the target sequences were synthesized by GeneCopoeia (Guangzhou, China). The following sequences were targeted to human ALKBH5, ATG5, ATG7, ULK1, Beclin1, and PI3KC3 (Phosphatidylinositol 3-kinase catalytic subunit type 3) small interfering RNA (siRNA): ALKBH5–1: 5′-GGAUAUGCUGCUGAUGAAATT-3′;ALKBH5–2: 5′-UCAGAUCGCCUGUCAGGAATT-3′;ATG7: 5′-CAGUGGAUCUAAAUCUCAAACUGAU-3′;ATG5: 5-CAAUCCCAUCCAGAGUUGCUUGUGA-3′;Beclin1: 5′-GCUGCCGUUAUACUGUUCU-3; ULK1: 5′-UGGUCAUGGAGUAUUGUAATT-3′;PI3KC3: 5′-GUGUGAUGAUAAGGAAUAU-3′ and NC (negative control) siRNA: 5′-UUCUUCGAAGGUGUCACGUTT-3′. The lentiviral vectors expressing shRNA targeting ALKBH5 (named LVRU6P-01 and LVRU6P-02) and the ALKBH5-lentiviral expression vector (named LV121-ALKBH5) were provided by GeneCopoeia. Also, we constructed pCMV5-*BCL-2* for the overexpression of Bcl-2 (B-cell lymphoma-2).

### Cell proliferation assay

Cell proliferation was assessed using the EdU assay as described previously [8]. The assay was performed using the Cell-Light™ EdU imaging detection kit according to the manufacturer’s instructions (Ruibo Biotechnology, Guangzhou, China).

### In vivo tumor xenograft study

The procedures for animal experiments were approved by the Committee on the Use and Care on Animals (Chongqing Medical University, Chongqing, China) and performed in accordance with the institutional guidelines. SKOV3 or A2780 cells were infected with the indicated lentiviral vectors and injected (5 × 10^6^ cells/mouse in 200 μL volume) subcutaneously into the left armpit of 6-week-old BALB/c nude mice. After 21 days, the animals were sacrificed to confirm the presence of tumors and weigh the established tumors [9].

### Matrigel invasion assays

The invasion ability of ovarian cancer SKOV3 and A2780 cells was evaluated by Matrigel invasion assay. The upper side of the 8-mm pore and the 6.5-mm polycarbonate transwell filter (Corning Inc., CLS3422) chamber were uniformly coated with Matrigel basement membrane matrix (BD Biosciences, 356,234) for 2 h at 37 °C. An equivalent of 5 × 10^4^ infected cells (SKOV3 cells infected with LVRU6P-NC, LVRU6P-01, or LVRU6P-02 and A2780 cells infected with LV121-NC or LV121-ALKBH5) were seeded into the top chamber on a transwell filter (in triplicate) and incubated in a serum-free medium for 48 h. The invasive cells on the lower side of the filter were fixed with 4% paraformaldehyde, stained with 0.5% crystal violet (Beyotime Institute of Biotechnology, C0121), and counted using a microscope. A total of five random fields were examined for each transwell filter and images captured at 200X magnification.

### Detection of protein expression by Western blotting (WB)

The expressions of ALKBH5, ATG7 (Autophagy related 7), ATG5(Autophagy related 5), Beclin1, ULK1 (Serine/Threonine-Protein Kinase ULK1), PI3KC3, p-MTOR (Mammalian target of rapamycin), and Actin proteins were analyzed by WB. The primary antibodies included monoclonal rabbit anti-ALKBH5 (Abcam, ab234528), rabbit monoclonal to ATG7 (Abcam, ab52472), rabbit monoclonal to ATG5 (Abcam, ab109490), rabbit polyclonal to Beclin1 (Abcam, ab62557), rabbit polyclonal to ULK1 (Abcam, ab203207), rabbit monoclonal to PI3KC3 (Abcam, ab40776), rabbit monoclonal to p-mTOR (Abcam, ab109268). Monoclonal mouse anti-β-actin (Abcam, ab8226) was used as an internal control to evaluate the band density on a gel imaging system.

### Quantitative real-time polymerase chain reaction (RT-qPCR)

Total RNA was extracted using a high-purity Total RNA Rapid Extraction Kit (Bioteke Corporation, RP1201) according to the manufacturer’s instruction. cDNA was synthesized using the iSCRIPT cDNA synthesis kit (Bio-Rad Laboratories, 4,106,228). The primers used for amplifying *ALKBH5* and *β-actin* were synthesized by GeneCopoeia.

### Estimation of total m6A and m6A^+^ BCL-2 mRNA levels

The total m6A content was measured in 200 ng aliquots of total RNA using an m6A RNA methylation quantification kit (cat. no. P-9005; Epigentek) according to the manufacturer’s instructions. To measure m6A^+^ *BCL-2* mRNA levels, m6A immunoprecipitation was performed as described previously. m6A^+^ RNA was purified by phenol/chloroform extraction and analyzed by RT-qPCR.

### Dual-luciferase reporter gene assay

The luciferase coding sequence *BCL-2* 3′-UTR reporter and luciferase coding sequence reporter were purchased from Genepharma. Cells were seeded in a 24-well plate and transfected with luciferase-*BCL-2* 3′-UTR reporter or luciferase reporter. After 48 h, the luciferase activity was measured using the Dual-Luciferase Reporter Assay System (Promega Corporation, E1910) according to the manufacturer’s instructions. Adenine-to-thymine mutation of the luciferase-*BCL-2* 3′-UTR reporter plasmid was effectuated using the Q5 Site-Directed Mutagenesis Kit (New England Biolabs) according to the manufacturer’s instructions. All experiments were performed at least three times.

### Statistical analysis

All statistical analyses were performed using SPSS software, version 17.0 (Chicago, IL, USA). Each experiment was performed in triplicate. The statistical analysis was performed using Student’s t-test or analysis of variance (ANOVA). The chi-square test was used to compare the association between the ALKBH5 overexpression and the clinicopathological variables of the ovarian cancer samples. Data were presented as mean ± standard deviation (mean ± SD). Statistical significance was defined as a *P*-value < 0.05.

## Results

### Aberrant expression of ALKBH5 in epithelial ovarian carcinoma tissues

**Fig. 1:**
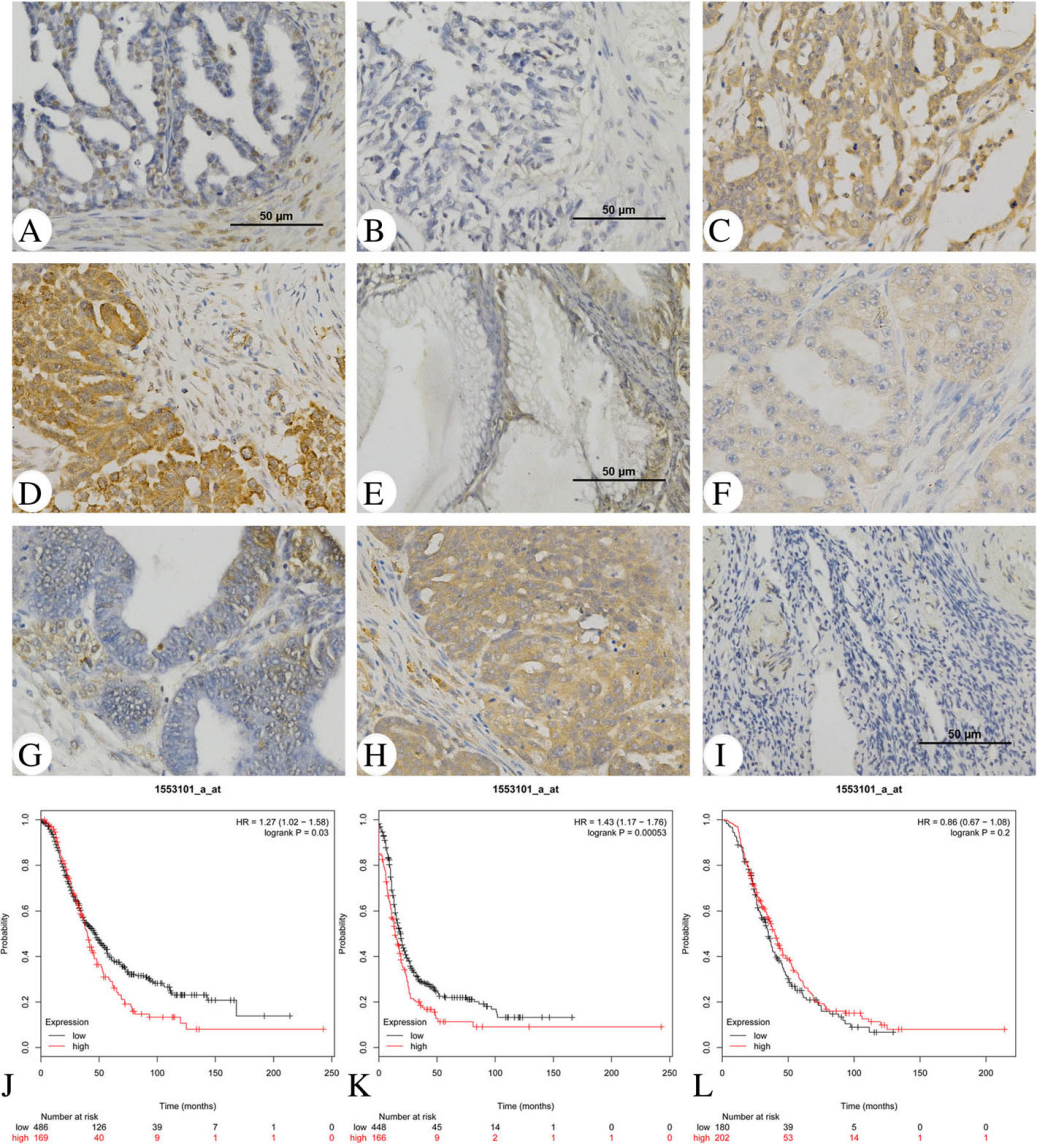
IHC analysis of ALKBH5 expression in epithelial ovarian cancer. ALKBH5 was predominantly localized in the (**d**) plasma membrane and (**a, c, h**) cytoplasm. The expression of ALKBH5 in different types of ovarian cancer samples. **a** Serous papillary adenocarcinoma (stage I); **b** Serous papillary adenocarcinoma (stage I); **c** Serous papillary adenocarcinoma (stage IV); **d** Serous papillary adenocarcinoma (stage IIIC); **e** Mucinous adenocarcinoma (stage IB); **f** Clear cell carcinoma (stage I); **g** Endometrioid adenocarcinoma (stage IB); **h** Endometrioid adenocarcinoma (stage IIIC); **i** Normal ovarian tissue. Original magnification, 200X. **j** PFS curves for ovarian cancer patients. **k** OS curves for ovarian cancer patients. **l** PPS curves for ovarian cancer patients. *P* < 0.05 was considered to be a statistically significant difference

The expression pattern of ALKBH5 in epithelial ovarian cancer is not yet fully elucidated. The present study examined the expression of ALKBH5 in normal ovary tissues and ovarian cancer tissue using IHC. ALKBH5 was predominantly localized on the plasma membrane and in the cytoplasm (Fig. 1a, b, and e). ALKBH5 expression was significantly higher in the epithelial ovarian cancer samples than in the normal ovary tissues (*P* < 0.05; Table 1). To investigate the correlation of ALKBH5 expression with the cancer type and cancer stage, all cancer samples were grouped into histological types (clear cell carcinoma, endometrioid adenocarcinoma, mucinous adenocarcinoma, and serous papillary adenocarcinoma). The expression of ALKBH5 was significantly higher in cancer samples in advanced stages (stage III/IV) as compared to those in the early stages (stage I/II) disease (*P* < 0.05). Furthermore, the staining intensity was significantly correlated with the tumor grade (grades 2–3 vs. 1, *P* < 0.05). However, the association between ALKBH5 expression and age was not significant (*P* > 0.05; Table 1). Subsequently, the ALKBH5 genes were examined with respect to progression-free survival (PFS), overall survival (OS), and post-progression survival (PPS) using the KM Plotter Online Tool (http://www.kmplot.com). The low expression of ALKBH5 mRNA was significantly correlated with PFS and OS, but that with PPS was not significant in any of the ovarian cancer patients (Fig. 1j-l).

**Table 1:** Association of ALKBH5 expression with clinicopathological characteristics in 158 patients of EOC

	No. of patients	ALKBH5 expression		*P* *value*
	(*n* = 158)	Low no.(%)	High no.(%)	
Characteristics				
Age (years)				> 0.05
< 50	83	36 (43.37%)	47 (56.63%)	
≥ 50	75	35 (46.67%)	40 (53.33%)	
Normal ovarian	20	17 (85%)	3 (15%)	< 0.05
Cancer tissues	138	77 (55.80)	61 (44.20%)	
FIGO stage				
I/II	103	67 (65.05%)	36 (34.95%)	< 0.05
III/IV	35	10 (28.57%)	25 (71.43%)	
Grade				
1	30	25 (83.33%)	5 (16.67%)	
2	36	21 (58.33%)	15 (41.67%)	
3	72	31 (43.06%)	41 (56.94%)	
		Grade 2–3 versus 1		< 0.05
Tumor type				
Serous	120	64 (53.33%)	56 (46.67%)	
Mucinous	14	11 (78.57%)	3 (21.43%)	
Endometrioid	4	2 (50.00%)	2 (50.00%)	
		Serous versus non-serous		
				> 0.05

### ALKBH5 regulated autophagy flux

**Fig. 2:**
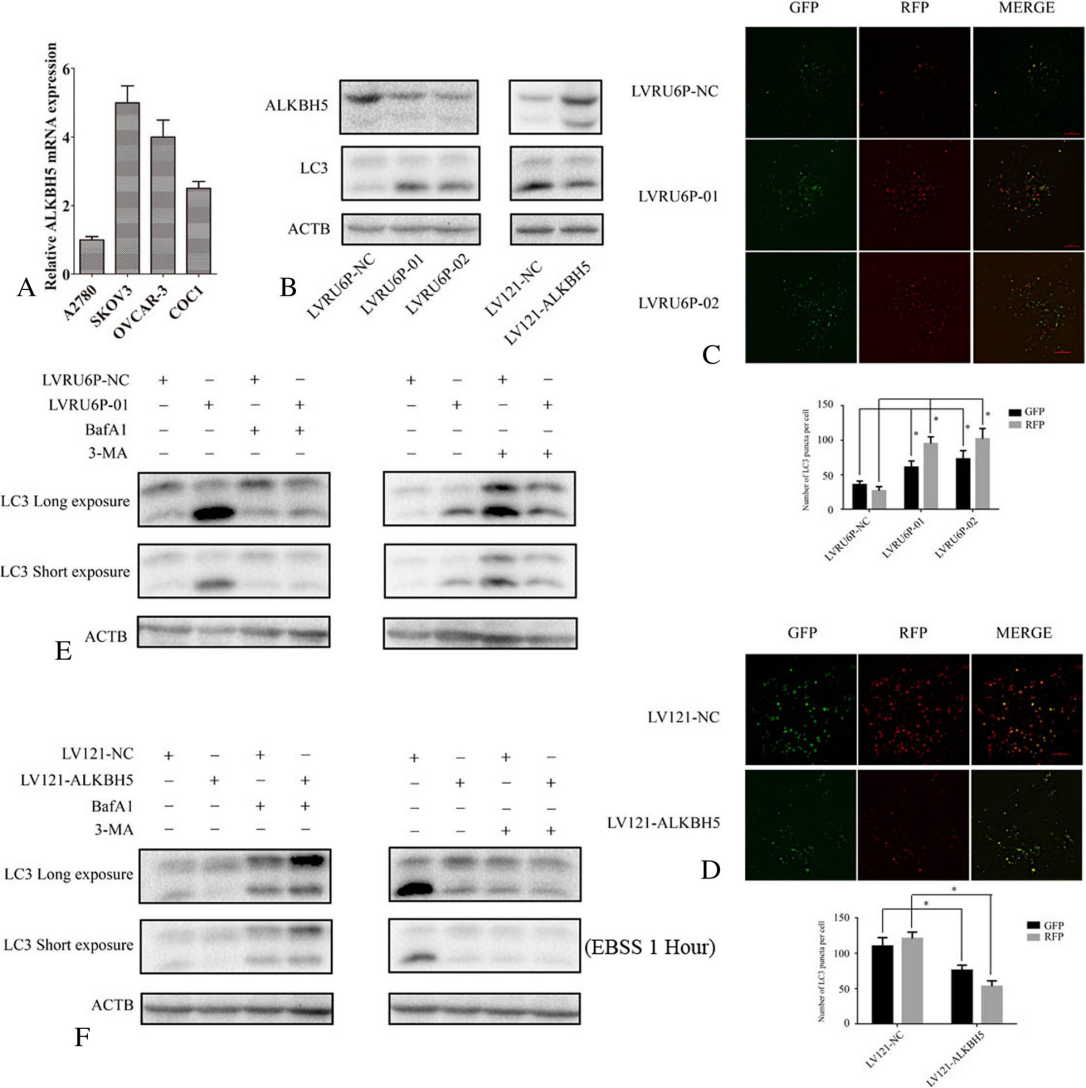
ALKBH5 regulated the autophagy flux. **a** The expression of *ALKBH5* mRNA in ovarian cancer cells was determined using qPCR. **b** The expression of LC3-II and ALKBH5 was detected using WB. **c** SKOV3 cells infected with LVRU6P-01, LVRU6P-02, or LVRU6P-NC. After 48 h, puromycin was added at a concentration of 2.5 mg/mL. Then, cells were transfected with a plasmid encoding mRFP-GFP-LC3 that was distributed in SKOV3 cells and analyzed by confocal microscopy after 48 h post-transfection. The LC3 puncta were quantified using Image Pro-Plus 6.0 software. All experiments were repeated three times. The bottom panel indicates the quantification of LC3 punctate staining. **d** A2780 cells were infected with LV121-NC or LV121-ALKBH5. After 48 h, puromycin was added at a concentration of 2.5 mg/mL. Then, cells were transfected with a plasmid encoding mRFP-GFP-LC3. The distribution of mRFP-GFP-LC3 in A2780 cells was analyzed by confocal microscopy after 48 h post-transfection. The LC3 puncta were quantified using Image Pro-Plus 6.0 software. All experiments were repeated three times. The bottom panel indicates the quantification of LC3 punctate staining. **e** and **f** The expression of LC3-II was detected by WB. Error bars represent the standard error. * indicated *P* < 0.05. Scale bar: 5 μm

To select the suitable cell lines for functional study, the *ALKBH5* expression was detected by qPCR and results showed that it was higher in the SKOV3 cell line than that in the A2780 cell line (Fig. 2a). Thus, SKOV3 cells were chosen for silencing *ALKBH5,* and A2780 cells were utilized for exogenous expression to investigate the ALKBH5 functions. The expression of MALKBH5 was suppressed in LVRU6P-01 or LVRU6P-02-infected SKOV3 cells as compared to LVRU6P-NC SKOV3 cells (Fig. 2b). Silencing or ectopic expression ALKBH5 did not change the apoptosis rate (data not shown). So, we determined whether ALKBH5 regulated the autophagy in ovarian cancer. We observed the expression of LC3-II was elevated in LVRU6P-01 or LVRU6P-02-infected SKOV3 cells as compared to LVRU6P-NC SKOV3 cells. Conversely, the expression of LC3-II was decreased in A2780 cells-infected LV121-ALKBH5 than that of LV121-NC (Fig. 2b).

The increased expression of LC3-II might be attributed to autophagy activation or reduced autophagosome turnover due to the defects in the formation of autolysosomes. Therefore, the autophagy inhibitors, 3-MA (3-methyladenine) and bafilomycin A1 (BafA1) were used to treat the cells individually. The effect of *ALKBH5* knockdown on LC3-II accumulation was compromised in the presence of 3-MA that inhibited the activity of class III phosphatidylinositol 3-kinase. Conversely, after BafA1 was added, the increased expression of LC3-II was observed as a result of *ALKBH5* knockdown (Fig. 2e). Ectopic expression of ALKHB5 inhibited the expression of LC3-II. This regulation was impeded in the presence of 3-MA (Fig. 2f). These results indicated that ALKBH5 regulated the initiation of autophagy but not the maturation.

Furthermore, we used the mRFP-GFP-LC3 reporter to detect the autophagy flux. In the lysosome lumen, the GFP signal was sensitive to the acidic condition, whereas the RFP signal was stable. Thus, this form of LC3 displayed only red fluorescence when localized in autolysosomes. The red puncta were increased in SKOV3 cells-infected LVRU6P-01 or LVRU6P-02 than that of LVRU6P-NC. However, the red puncta were decreased in A2780 cells-infected LV121-ALKBH5 than that of LV121-NC. These results indicated that *ALKBH5* knockdown activated autophagy (Fig. 2c and d, Additional file 1: Figure S1 and Additional file 2: Figure S2).

### ALKBH5 mediated autophagy was associated with the cellular proliferation and migration

**Fig. 3:**
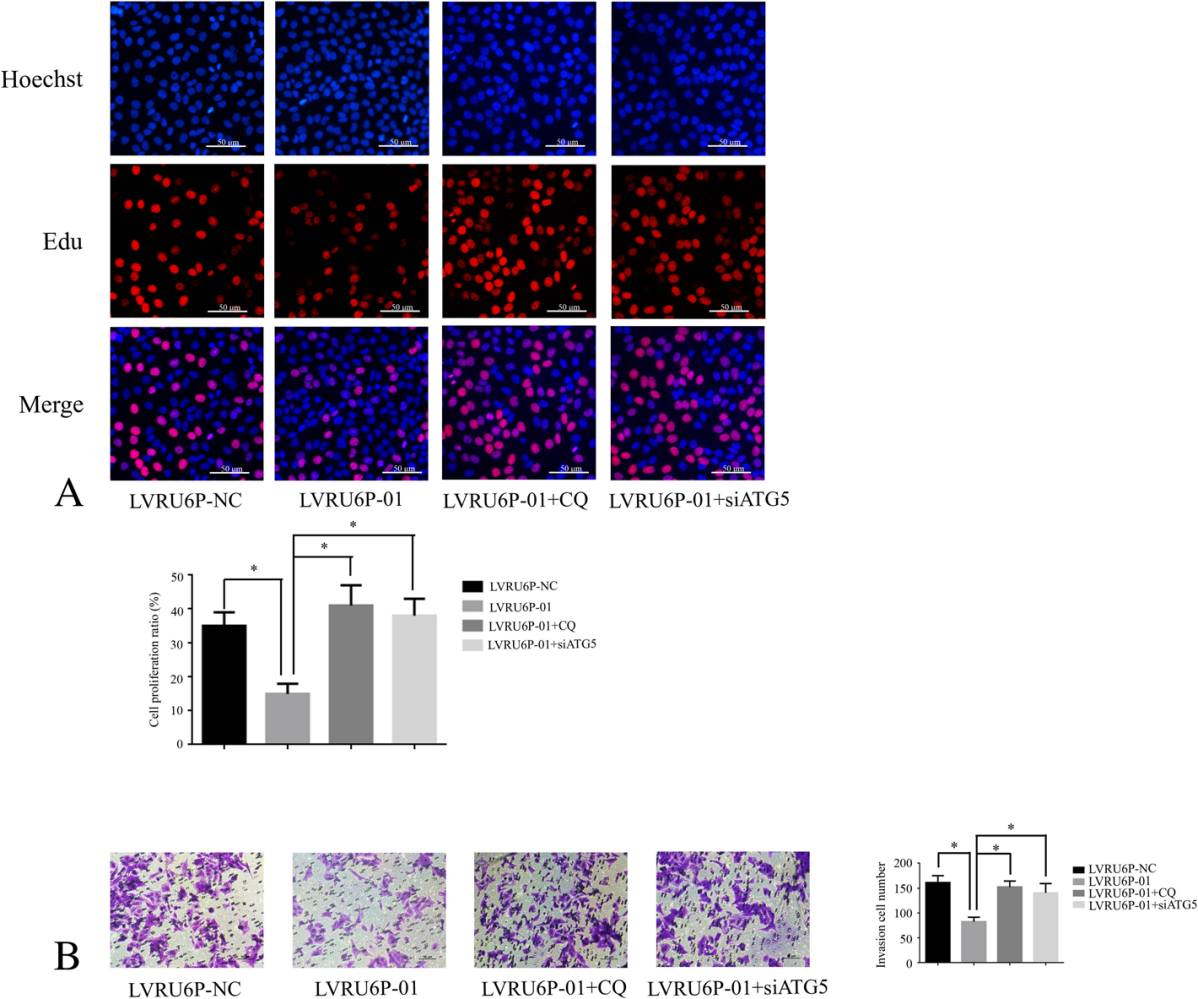
ALKBH5 regulates the cellular proliferation and migration. **a** SKOV3 cells were infected with LVRU6P-01, LVRU6P-01 + CQ, LVRU6P-01 + siATG5, or LVRU6P-NC. After 48 h, puromycin was added at a concentration of 2.5 mg/mL. The cell proliferation was assessed by EdU assay. Original magnification, 200X. **b** SKOV3 cells were infected with LVRU6P-01, LVRU6P-01 + CQ, LVRU6P-01 + siATG5, or LVRU6P-NC. After 48 h, puromycin was added at a concentration of 2.5 mg/mL. After 72 h, the invasion assays were performed. Error bars represent standard error. * indicated *P* < 0.05. Scale bar: 50 μm

Autophagy has been demonstrated as a tumor suppressor in some cancer types. Thus, we test effect of silencing ALKBH5 induced autophagy on cellular proliferation and migration. We found that proliferation and migration ability in SKOV3 cells infected by LVRU6P-01 were decreased. When added CQ or silencing ATG5, the proliferation and migration ability was elevated in SKOV3 cells (Fig. 3a-b).

### ALKBH5-regulated autophagy was required for ULK1, PIK3C3, BECN1, ATG5 and ATG7

**Fig. 4:**
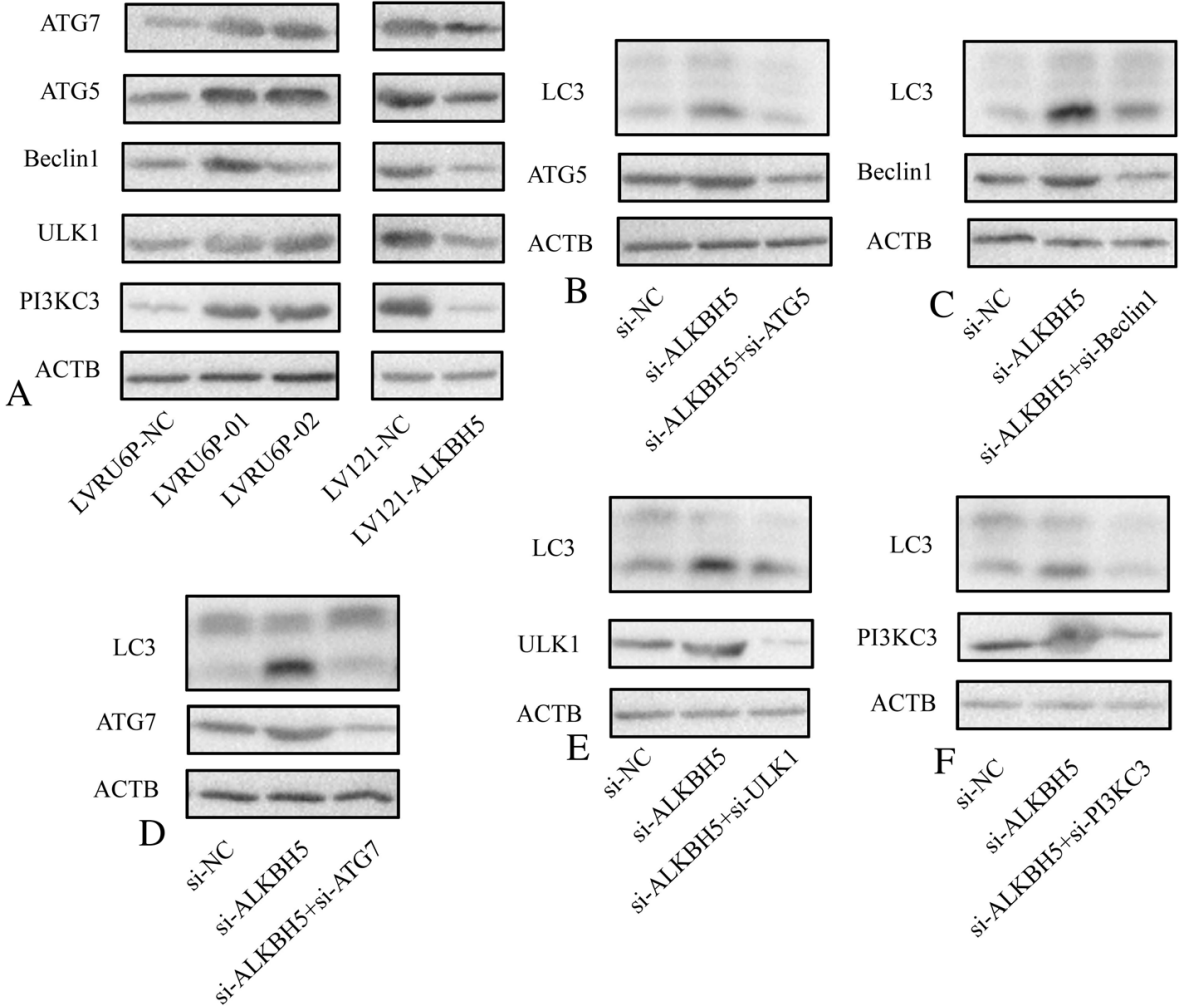
ALKBH5-regulated autophagy acts upstream of ULK1 and the PIK3C3-BECN1 complex. **a** SKOV3 cells were infected with LVRU6P-01, LVRU6P-02, or LVRU6P-NC. A2780 cells were infected with LV121-NC or LV121-ALKBH5. After 48 h, puromycin was added at a concentration of 2.5 mg/mL. After 72 h, the expression of ATG5, ATG7, Beclin1, Ulk1, and PI3KC3 was detected by WB. **b** SKOV3 cells were infected with LVRU6P-01 or LVRU6P-NC. After 48 h, puromycin was added at a concentration of 2.5 mg/mL. After 24 h, the LVRU6P-01-infected cells were transfected with ATG5 siRNAs. After 48 h, the expression of ATG5 and LC3-II was detected using WB. **c** SKOV3 cells were infected with LVRU6P-01 or LVRU6P-NC. After 48 h, puromycin was added at a concentration of 2.5 mg/mL. After 24 h, the LVRU6P-01 infected cells were transfected with Beclin1 siRNAs. After 48 h, the expression of Beclin1 and LC3-II was detected using WB. **d** SKOV3 cells were infected with LVRU6P-01 or LVRU6P-NC. After 48 h, puromycin was added at a concentration of 2.5 mg/mL. After 24 h, the LVRU6P-01 infected cells were transfected with ATG7 siRNAs. After 48 h, the expression of ATG7 and LC3-II was detected using WB. **e** SKOV3 cells were infected with LVRU6P-01 or LVRU6P-NC. After 48 h, puromycin was added at a concentration of 2.5 mg/mL. After 24 h, the LVRU6P-01 infected cells were transfected with Ulk1 siRNAs. After 48 h, the expression of Ulk1 and LC3-II was detected by WB. **f** SKOV3 cells were infected with LVRU6P-01 or LVRU6P-NC. After 48 h, puromycin was added at a concentration of 2.5 mg/mL. After 24 h, the LVRU6P-01-infected cells were transfected with PI3KC3 siRNAs. After 48 h, the expression of PI3KC3 and LC3-II was detected using WB

Next, we determined at which step the autophagosome formation is increased in the ALKBH5-knockdown cells. Strikingly, several key ATG molecules, including ATG5, ATG7, PIK3C3, BECN1, and ULK1 were analyzed. The expression of ATG5, ATG7, PIK3C3, BECN1, and ULK1 was increased in SKOV3 cells-infected LVRU6P-01 or LVRU6P-02 as compared to that infected LVRU6P-NC. On the other hand, the expression of ATG5, ATG7, PIK3C3, BECN1, and ULK1 was decreased in A2780 cells infected by LV121-ALKBH5 as compared to that with LV121-NC (Fig. 4a). ATG5, ATG7, PIK3C3, BECN1, or ULK1 knockdown reversed the accumulation of LC3B-II induced by siALKBH5 (Fig. 4b-f). These data implied that the activity of ATG5, ATG7, PIK3C3, BECN1, and ULK1 was required for *ALKBH5* silencing-induced autophagy.

### ALKBH5 regulated the cellular proliferation, migration, and autophagy through the EGFR-PI3K-AKT-mTOR pathway

**Fig. 5:**
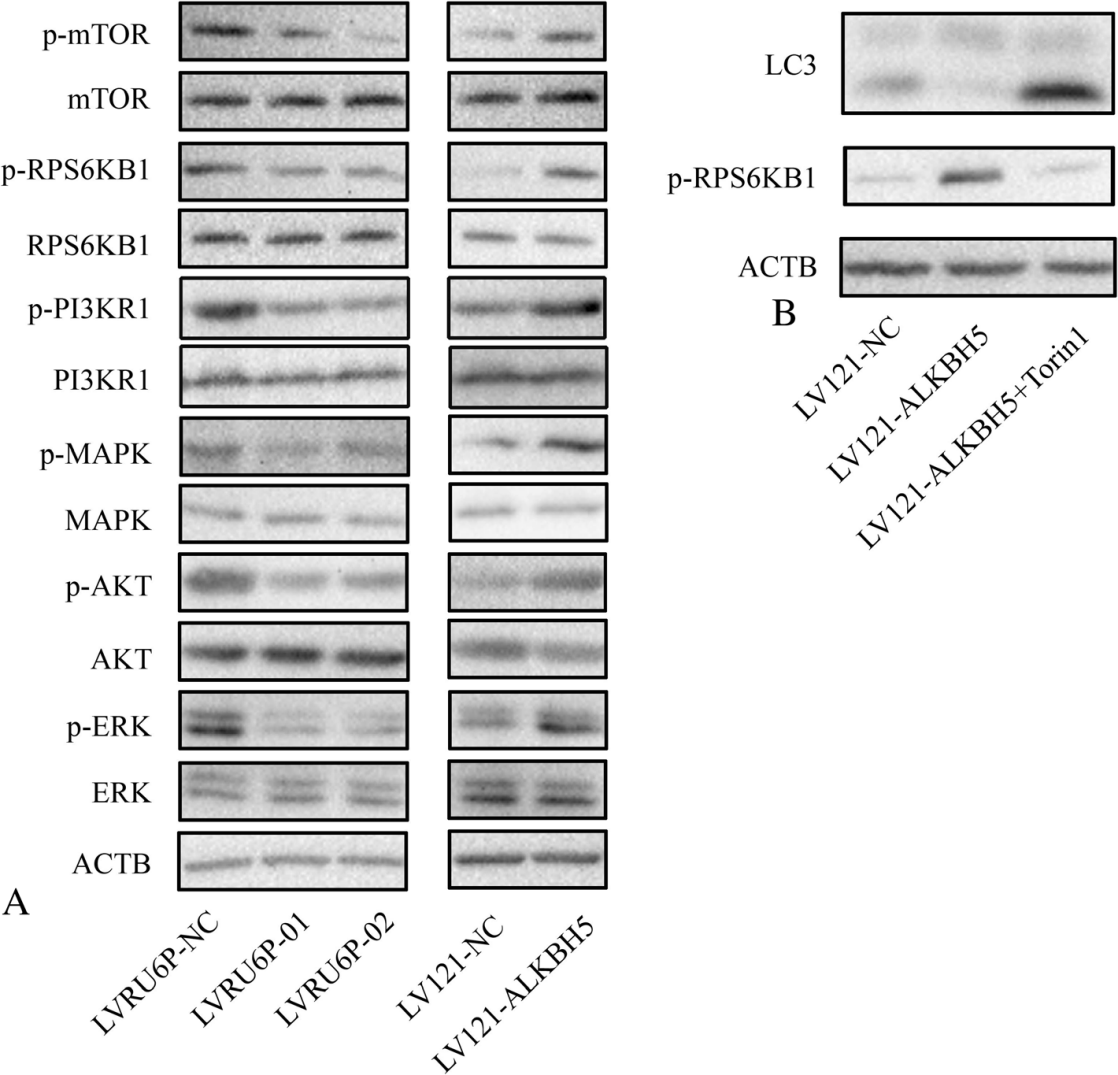
ALKBH5 regulated the cellular proliferation, migration, and autophagy through mTOR pathway. **a** SKOV3 cells were infected with LVRU6P-01, LVRU6P-02, or LVRU6P-NC. A2780 cells were infected with LV121-NC or LV121-ALKBH5. After 48 h, puromycin was added at a concentration of 2.5 mg/mL. After 72 h, the expression of mTOR pathway was detected by WB. **b** A2780 cells were infected with LV121-NC or LV121-ALKBH5. After 48 h, puromycin was added at a concentration of 2.5 mg/mL. After 24 h, Torin1 was added at a concentration of 10 μM. After 8 h, the expression of LC3-II and p-RPS6KB1 was detected by WB

The mTOR pathway is a key regulator in autophagy induced due to nutrient deprivation and stress. Interestingly, the mTOR pathway activity is inversely correlated with autophagy induction. Thus, we investigated whether ALKBH5 regulated this pathway and found that the expression of p-mTOR was decreased in SKOV3 cells infected with LVRU6P-01 or LVRU6P-02 as compared to that of SKOV3 cells infected with LVRU6P-NC. However, the expression of p-mTOR was increased in A2780 cells infected with LV121-ALKBH5 as compared to that of A2780 cells infected with LV121-NC. In addition, we observed that the expression of EGFR, PI3K and p-AKT was decreased after silencing ALKBH5. Conversely, the expression of EGFR, PI3K and p-AKT was increased after the overexpression of ALKBH5 (Fig. 5a). We also observed the expression of p-MAPK, and p-Erk1/2 was regulated by ALKBH5 (Fig. 5a).

Subsequently, whether ALKBH5 regulated autophagy via the mTOR pathway was determined. Torin1 is an inducer of autophagy and inhibitor of the mTOR pathway. It partially restored the suppressed expression of LC3-II in A2780 cells infected with LV121-ALKBH5 (Fig. 5b).

### ALKBH5 regulated the expression of EGFR dependent on HuR and miR-7

**Fig. 6:**
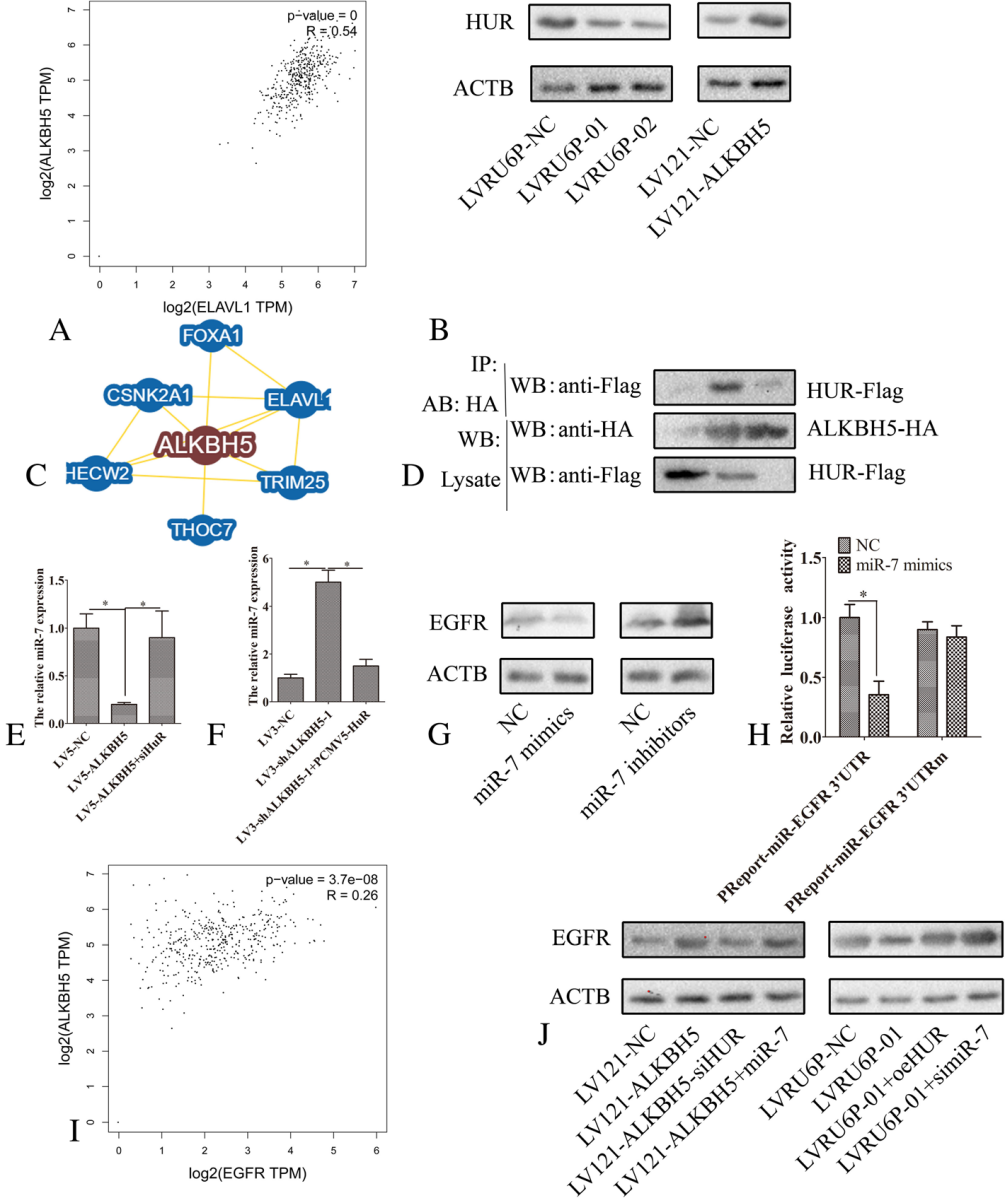
ALKBH5 regulated EGFR dependent on HuR and miR-7. **a** We analyzed the correlation between ALKBH5 and HuR using TCGA (http://gepia.cancer-pku.cn/). **b** SKOV3 cells were infected with LVRU6P-01, LVRU6P-02, or LVRU6P-NC. A2780 cells were infected with LV121-NC or LV121-ALKBH5. After 48 h, puromycin was added at a concentration of 2.5 mg/mL. After 72 h, the expression of HuR was detected by WB. **c** Bioinformatics prediction the interaction between HuR and ALKBH5 (https://thebiogrid.org/). **d** The interaction between HuR and ALKBH5 was confirmed using IP. **e**-**f** The expression of miR-7 was determined by qPCR. **g** The expression of EGFR was detected by WB. **h** Dual luciferase reporter assay was used to confirm the interaction between miR-7 and EGFR. **i** We analyzed the correlation between ALKBH5 and EGFR using TCGA (http://gepia.cancer-pku.cn/). **j**The expression of EGFR was determined by WB. Error bars represent the standard error. * indicated *P* < 0.05

A previous study found that ALKBH5 regulated the expression of HuR. First, we analyzed and established a positive correlation between ALKBH5 and HuR protein expression in ovarian cancer tissues through the TCGA database (http://gepia.cancer-pku.cn/index.html) (Fig. 6a). The silencing of ALKBH5 resulted in significantly decreased protein levels of HuR in ovarian cancer SKOV3 cells. The ectopic expression of ALKBH5 induced the expression of HuR in A2780 cells (Fig. 6b). Bioinformatics predicted that ALKBH5 interacted with HuR (Fig. 6c). We confirmed the physical interactions between HuR and ALKBH5 (Fig. 6d). These results indicated that ALKBH5 might be directly involved in regulating the stability of HuR target genes. Another previous study demonstrated that HuR repressed the miR-7 expression by regulating its processing. Thus, whether ALKBH5 regulates miR-7 expression in an HuR-dependent manner is yet to be elucidated. As expected, the ectopic expression of ALKBH5 inhibited the expression of miR-7 in A2780 cells; however, this regulation was inhibited by silencing HuR (Fig. 6e). Furthermore, silencing ALKBH5 elevated the expression of miR-7 in SKOV3 cells, which in turn, was inhibited by the ectopic expression of HuR (Fig. 6f). EGFR is shown to be a direct target of miR-7. The current study found that miR-7 mimics inhibited the protein level of EGFR, while miR-7 inhibitors elevated the level of EGFR protein (Fig. 6g). Dual luciferase reporter assay confirmed that EGFR is a direct target of miR-7 (Fig. 6h). Next, a positive correlation was established between ALKBH5 and EGFR protein expression in ovarian cancer tissues using the TCGA database (http://gepia.cancer-pku.cn/index.html) (Fig. 6i). The ectopic expression of ALKBH5 promoted the expression of EGFR in A2780 cells; however, this regulation was inhibited when silencing the HuR or ectopic expression of miR-7. Next, silencing ALKBH5 repressed the expression of EGFR in SKOV3 cells; however, this regulation was abolished when the ectopic expression of HuR silenced miR-7 (Fig. 6j). These data suggested that ALKBH5 physically interacted with HuR, inhibited miR-7, and promoted the EGFR expression.

### ALKBH5 enhances BCL-2 mRNA stability by catalyzing m6A demethylation and promoted the interaction between Bcl-2 and Beclin1

**Fig. 7:**
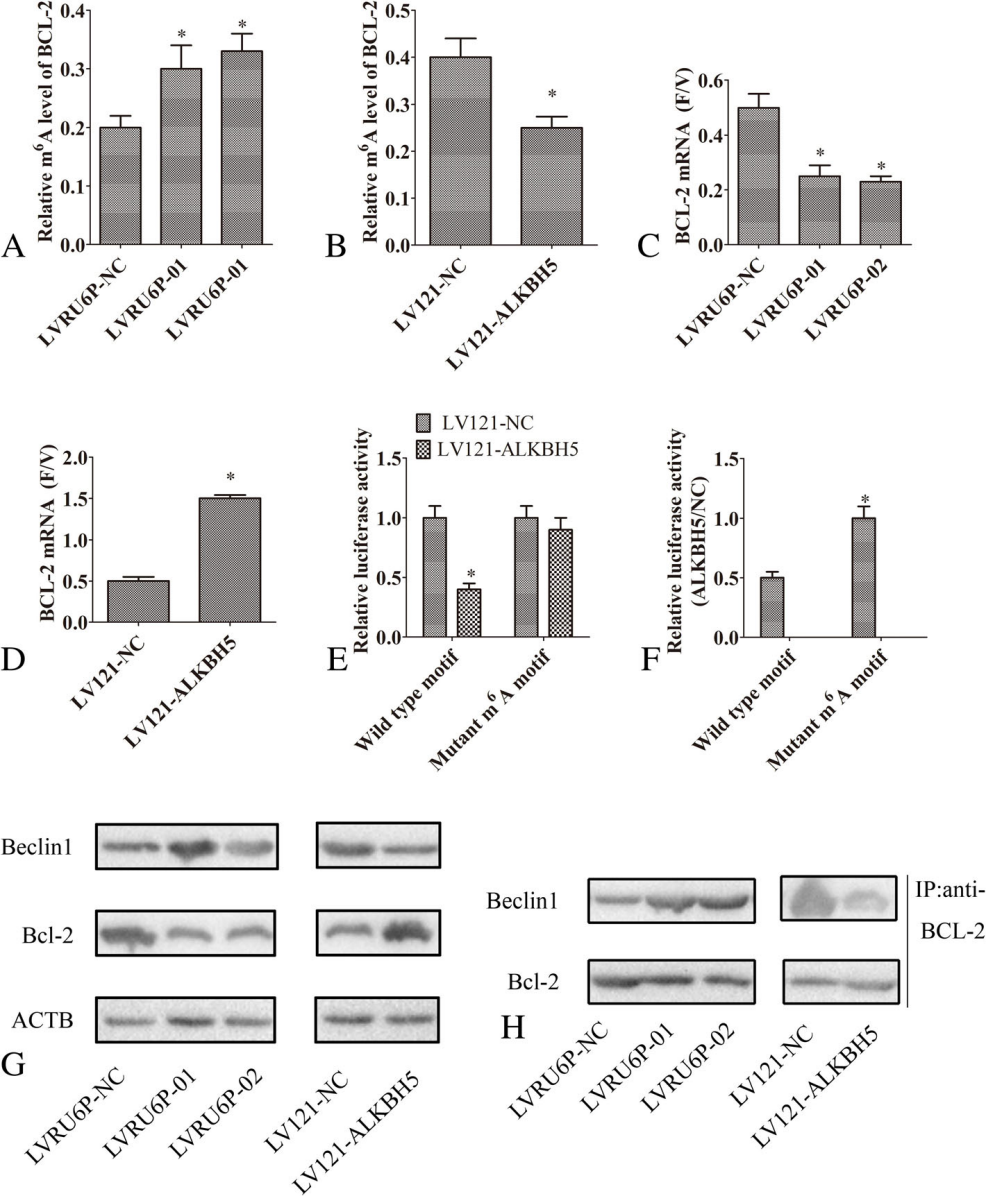
ALKBH5 enhanced *BCL-2* mRNA stability by catalyzing m6A demethylation and promoted the interaction between Bcl-2 and Beclin1. **a** Gene-specific m6A qPCR analysis of m6A level in mRNA transcripts of *ALKBH5* in SKOV3 cells infected with LVRU6P-01, LVRU6P-02, or LVRU6P-NC. **b** Gene-specific m6A qPCR analysis of m6A level in mRNA transcripts of *ALKBH5* in A2780 cells infected with LV121-NC or LV121-ALKBH5. **c** SKOV3 cells infected with LVRU6P-01, LVRU6P-02, or LVRU6P-NC. **d** A2780 cells were infected with LV121-NC or LV121-ALKBH5, followed by treatment with vehicle or flavopiridol for 6 h. The mRNA levels of *BCL-2* were measured by RT-qPCR, and the F/V ratio of *NANOG* mRNA was determined. **e** Relative luciferase activity of pMIR-REPORT-*BCL-2*-3′-UTR with either wild-type or mutant (A-to-T mutation) m6A sites after co-transfection with LV121-ALKBH5 or LV121-NC into A2780 cells. Firefly luciferase activity was measured and normalized to Renilla luciferase activity. **f** Luciferase assays were performed in A2780 cells transfected with wild-type or mutant luciferase-*BCL-2*-3′-UTR reporter. **g** SKOV3 cells were infected with LVRU6P-01, LVRU6P-02, or LVRU6P-NC. A2780 cells were infected with LV121-NC or LV121-ALKBH5. Then, the expression of Bcl-2 and Beclin1 was determined by WB. **h** SKOV3 cells were infected with LVRU6P-01, LVRU6P-02 m or LVRU6P-NC. A2780 cells were infected with LV121-NC or LV121-ALKBH5. Subsequently, the immunoprecipitation of endogenous Beclin1 with endogenous Bcl-2 was performed. Error bars represent the standard error. * indicated *P* < 0.05

Bioinformatics analysis (http://rna.sysu.edu.cn/rmbase/index.php) demonstrated that m6A modification influenced the *BCL-2* mRNA regulation. The gene-specific m^6^A qPCR assays revealed that the levels of m6A^+^ *BCL-2* mRNA were increased in ovarian cancer SKOV3 cells infected with LVRU6P-01 or LVRU6P-01 as compared to that of SKOV3 cells infected with LVRU6P-NC (Fig. 7a). However, the levels of m6A^+^ *BCL-2* mRNA were decreased in ovarian cancer A2780 cells infected with LV121-ALKBH5 as compared with that of A2780 cells infected with LV121-NC (Fig. 7b). To evaluate the stability of *BCL-2* mRNA, flavopiridol was used to inhibit the global mRNA transcription. The ratio of *BCL-2* mRNA in flavopiridol-treated cells relative to vehicle-treated cells (F/V ratio) was calculated. The *BCL-2* mRNA F/V ratio was decreased in ovarian cancer SKOV3 cells infected with LVRU6P-01 or LVRU6P-02 as compared to that of SKOV3 cells infected with LVRU6P-NC (Fig. 7c). However, the *BCL-2* mRNA F/V ratio was increased in ovarian cancer A2780 cells infected with LV121-ALKBH5 as compared to that of A2780 cells infected with LV121-NC (Fig. 7d).

To assess the requirement of the target mRNA m6A modifications for ALKBH5-mediated *BCL-2* regulation, we conducted a luciferase reporter assay. Consequently, the luciferase activity was decreased in ovarian cancer A2780 cells infected with LV121-ALKBH5 as compared to that of A2780 cells infected with LV121-NC, while mutations in the m6A sites abrogated the inhibition (Fig. 7e). Compared to the wild-type reporters, the mutation in the adenosine residue resulted in increased luciferase activity in ovarian cancer SKOV3 cells (Fig. 7f). These observations indicated that the mutation prevented methylation, and thus, increased the stability of the luciferase-*BCL-2* 3′-UTR fusion mRNA. These results suggested that ALKBH5 increased the *BCL-2* mRNA demethylation and stabilization.

ALKBH5 can regulate the m6A demethylation of the mRNA. *BCL-2* inhibited the autophagy through the Beclin1-Bcl-2 complex. Thus, we speculated that ALKBH5 regulated the Beclin1-Bcl-2 interaction. Strikingly, silencing ALKBH5 inhibited the interaction between Beclin1 and *BCL-2*. However, the overexpression of ALKBH5 promoted the interaction between Beclin1 and *BCL-2*.

### ALKBH5 regulated ovarian cancer proliferation, invasion, and autophagy flux through BCL-2

**Fig. 8:**
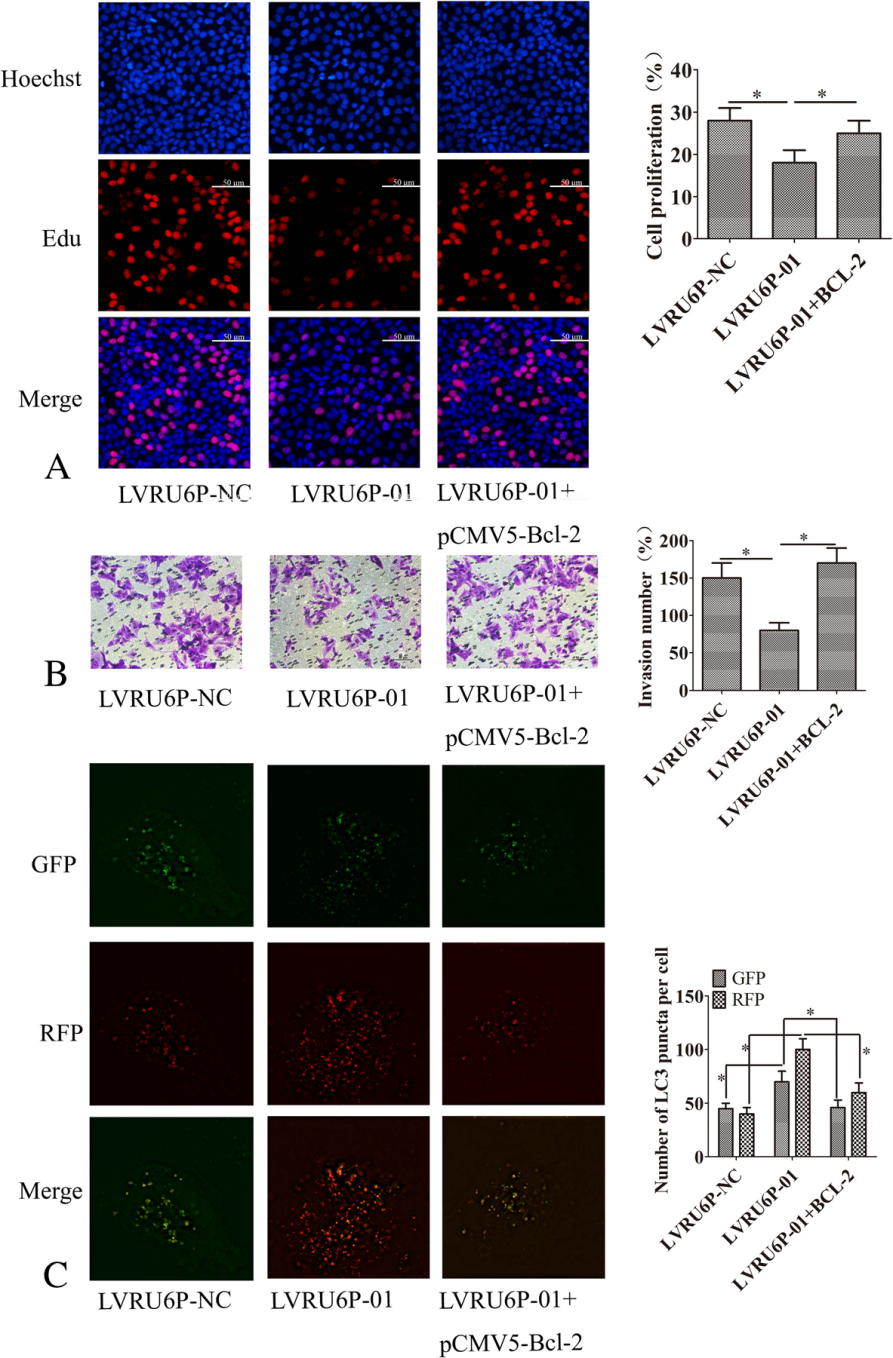
ALKBH5 regulated ovarian cancer proliferation, invasion, and autophagy flux through Bcl-2. **a** SKOV3 cells were infected with LVRU6P-01 or LVRU6P-NC. After 48 h, puromycin was added at a concentration of 2.5 mg/mL. After 24 h, the LVRU6P-01-infected cells were transfected with pCMV5-Bcl-2. After 48 h, the cell proliferation was assessed by EdU assay. Original magnification, 200X. **b** SKOV3 cells were infected with LVRU6P-01 or LVRU6P-NC. After 48 h, puromycin was added at a concentration of 2.5 mg/mL. After 24 h, the LVRU6P-01-infected cells were transfected with pCMV5-Bcl-2. After 48 h, the invasion assays were performed. **c** SKOV3 cells were infected with LVRU6P-01 or LVRU6P-NC. After 48 h, puromycin was added at a concentration of 2.5 mg/mL. After 24 h, the LVRU6P-01-infected cells were co-transfected with pCMV5-Bcl-2 and a plasmid encoding mRFP-GFP-LC3. mRFP-GFP-LC3 distribution in SKOV3 cells was analyzed by confocal microscopy after 48 h post-transfection. The LC3 puncta were quantified using Image Pro-Plus 6.0 software. All experiments were repeated three times. The right panel indicates the quantification of LC3 punctate staining. Error bars represent the standard error. *indicated *P* < 0.05. Scale bar: 50 μm

Silencing ALKBH5 inhibited the proliferation, migration and enhanced the autophagy in ovarian cancer SKOV3 cells. These phenotypes were restored when ectopic expression of *BCL-2* (Fig. 8a-c). These data indicated that ALKBH5 regulated ovarian cancer proliferation, invasion, and autophagy flux through *BCL-2*.

### ALKBH5 promoted xenograft tumor growth

**Fig. 9:**
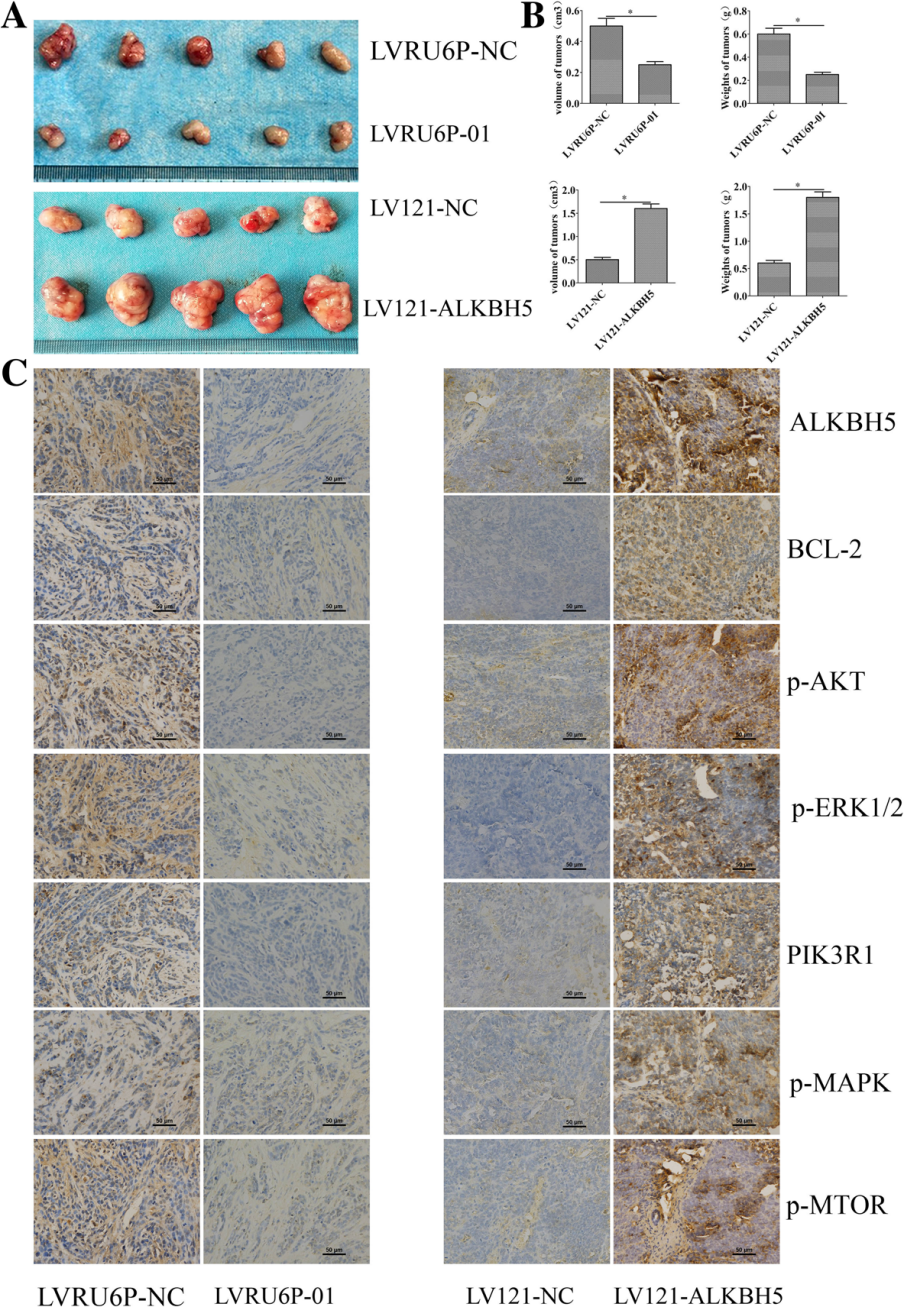
ALKBH5 regulated tumorigenesis in nude mice model. **a**, **b** Mean tumor volume and weight on day 21 after tumor cell injection. LVRU6P-01- or LVRU6P-NC-infected SKOV3 cells were implanted subcutaneously into the left armpit. A2780 cells infected with LV121-NC or LV121-ALKBH5 were implanted subcutaneously into the left armpit. **c** IHC of ALKBH5, Bcl-2, p-AKT, p-ERK1/2, PIK3R1, p-MAPK, and p-mTOR expression was analyzed on tumor xenografts. Representative images are shown (original magnification 200X). *indicated *P* < 0.05. Scale bar: 50 μm

The role of ALKBH5 in tumor formation of ovarian cancer cells, SKOV3 and A2780, was determined using an animal model. The average weight and volume of tumors were significantly lower in the LVRU6P-01-infected group than that in the LVRU6P-NC-infected group. The expression of ALKBH5, p-mTOR, p-ERK1/2, p-AKT, p-MAPK, PIK3R1, and Bcl-2 in tumors from the LV3-shALKBH5–1-infected group was lower than that in the LV3-NC-infected group. Conversely, the average weight and volume of the tumors were significantly higher in the LV121-ALKBH5-infected group than that in the LV121-NC-infected group. The expression of ALKBH5, p-mTOR, p-ERK1/2, p-AKT, p-MAPK, PIK3R1, and Bcl-2 in tumors from the LV121-ALKBH5-infected group was higher than that in the LV121-NC-infected group (Fig. 9a-b). These data showed that ALKBH5 promoted the tumor formation in vivo.

## Discussion

In the present study, we observed an increased expression of ALKBH5 in epithelial ovarian cancer tissues. We also found that the expression of ALKBH5 was correlated with the tumor stage and histological grades. Ovarian cancer patients with high expression of ALKBH5 had a poor OS and PFS. In addition, the upregulation of ALKBH5 expression promoted the proliferation, invasion, and autophagy in epithelial ovarian cancer cells. These data suggested that an elevated level of ALKBH5 promoted an aggressive behavior, indicating that the molecule can be a novel prognostic biomarker and a therapeutic target for the treatment of epithelial ovarian cancer. Furthermore, our data demonstrated that ALKBH5 promotes the interaction between Bcl-2 and Beclin1 by enhancing the *BCL-2* mRNA stability via m6A demethylation catalysis. ALKBH5 also regulates the ERK/MAPK and PI3K/AKT/mTOR pathways.

Although the role of autophagy in cancer is controversial, some studies demonstrated that autophagy could serve as a tumor suppressive mechanism. Some well-characterized tumor suppressors, such as PTEN and TSC1/2, negatively regulate the protein kinase mTOR and induce autophagy [8, 10–14]. In this study, ALKBH5 was found to promote proliferation and invasion but inhibit autophagy. Thus, *ALKBH5* may serve as a tumor-promoted gene.

Nevertheless, the role of autophagy in tumors is controversial. Some studies suggest that autophagy promotes tumor proliferation, invasion, and metastasis. However, some studies found that autophagy inhibits the malignant biological behavior of tumors [15, 16]. PBK promotes the metastasis of serous ovarian carcinoma and confers cisplatin resistance [17]. OGT was downregulated in ovarian cancer, which in turn, promotes cisplatin resistance by inducing autophagy [18]. The previous studies found that MARCH5 and PKP3 regulated the autophagy and invasion of ovarian cancer [7, 19]. Herein, we found that ALKBH5 inhibited autophagy and promoted proliferation and invasion of ovarian cancer. Next, silencing ALKBH5 induced autophagy. However, when autophagy was suppressed, the proliferation and migration ability was elevated. These data indicated that the inhibition of autophagy by ALKBH5 promoted the malignant behavior of ovarian cancer.

The mTOR signaling pathway is critical for the regulation of autophagy [11, 14]. The present study demonstrated that the phosphorylation levels of mTOR and RPS6KB1 were decreased in ALKBH5-silenced cells but increased in ALKBH5-overexpressing cells. However, this regulation was partially restored upon addition of the mTOR inhibitor. The PI3K-AKT and MAPK-ERK signals were upstream of mTOR, which activated TOR and consequently inhibited the autophagy. Furthermore, silencing the *ALKBH5* gene inhibited p-AKT, p-MAPK, and p-ERK, while the ectopic expression of ALKBH5 promoted the expression of p-AKT, p-MAPK, and p-ERK. These results indicated that ALKBH5 regulated autophagy through the mTOR pathway.

m6A is one of the most common RNA modifications in eukaryotes [20–22]. As an m6A eraser, ALKBH5 specifically removes m6A from the target mRNAs [2, 23]. In breast cancer, hypoxia elevated the expression of ALKBH5, and then, demethylated *NANOG* mRNA. An increased NANOG mRNA and protein expression promote the breast cancer stem cell phenotype [4, 5]. ALKBH5 is highly expressed in GSCs. The knockdown of ALKBH5 inhibited the proliferation of patient-derived GSCs. ALKBH5 demethylates FOXM1 nascent transcripts, resulting in enhanced FOXM1 expression [6]. In the present study, silencing ALKBH5 suppressed the protein and mRNA level of *BCL-2*. The overexpression of ALKBH5 elevated the expression of *BCL-2*. Also, the overexpression of ALKBH5 potentiated the decrease in m6A^+^ *BCL-2* mRNA levels. These data indicated that ALKBH5 regulated the expression of *BCL-2* mediated by m6A modification of *BCL-2* mRNA.

Beclin1 and Bcl-2 act as a switch between autophagy and apoptosis. Bcl-2 is a main anti-apoptotic protein of the Bcl family that interacts with Beclin1. Bcl-2 was demonstrated to inhibit autophagy by binding to Beclin1. Thus, promoting the binding of Bcl-2 and Beclin1 may be a mechanism underlying the inhibition of autophagy [24–26]. Conversely, the dissociation of Bcl-2 from Beclin1 might be critical for activating autophagy. Herein, we found that silencing ALKBH5 inhibited the *BCL-2* expression but increased that of Beclin1. The ectopic expression of ALKBH5 increased the expression of *BCL-2* but suppressed that of Beclin1. Also, we found that ectopic expression of ALKBH5 promoted the binding between *BCL-2* and Beclin1. These results suggested that ALKBH5 inhibited autophagy mediated by the interaction between Bcl-2 and Beclin 1.

## Conclusion

This study confirms that *ALKBH5* is a tumor-promoting gene in epithelial ovarian cancer, which is involved in the mTOR pathway and *BCL-2*-Beclin1 complex. Overall, the present study identified ALKBH5 as a candidate oncogene in epithelial ovarian cancer and a potential target for ovarian cancer therapy.

## Additional files

Additional file 1: **Figure S1.** Silencing ALKBH5 regulated autophagy flux. (TIF 2312 kb)

Additional file 2: **Figure S2.** Overexpression of ALKBH5 regulated autophagy flux. (TIF 1802 kb)

## Abbreviations

ATG5: Autophagy related 5
ATG7: Autophagy related 7
BCL-2: B-cell lymphoma-2
EGFR: Epidermal growth factor receptor
miR-7: microRNA 7
MTOR: Mammalian target of rapamycin
PI3KC3: Phosphatidylinositol 3-kinase catalytic subunit type 3
ULK1: Serine/Threonine-Protein Kinase ULK1
ULK1: Serine/threonine-protein kinase ULK1
